# Acute Transverse Myelitis Complicating the Third Trimester of Pregnancy: A Rare Case of Progressive Neurological Recovery and Favorable Obstetric Outcome

**DOI:** 10.7759/cureus.116898

**Published:** 2026-09-25

**Authors:** Aboubakr Benjilany, Saad Benali, Mly Mehdi Elhassani, Kouach Jaouad

**Affiliations:** 1 Department of Obstetrics and Gynecology, Military Hospital of Instruction Mohamed V, Rabat, MAR

**Keywords:** corticosteroids, myelopathy, parainfectious myelitis, pregnancy, tetraparesis, transverse myelitis

## Abstract

Transverse myelitis (TM) is a rare immune-mediated inflammatory disorder of the spinal cord. It can result in sensory, motor, and autonomic deficits of varying severity. Its occurrence during pregnancy is uncommon and presents specific diagnostic, therapeutic, obstetric, and anesthetic challenges.

We report the case of a 24-year-old para 2 woman who developed an acute febrile spinal cord syndrome at 30 weeks of gestation, characterized by rapidly progressive tetraparesis, sensory impairment, and sphincter dysfunction. Neurological deficits progressed to a nadir within the first 24 hours. Spinal magnetic resonance imaging performed on the first day demonstrated an extensive T2-hyperintense lesion extending from C5 to T1, consistent with acute TM, with no evidence of spinal cord compression. Cerebrospinal fluid analysis showed lymphocytic pleocytosis. Brain MRI was normal, anti-AQP4 and anti-MOG antibodies were negative, and no specific infectious agent was identified. A parainfectious etiology was suspected but could not be definitively established. The patient received high-dose intravenous methylprednisolone, initially combined with empirical anti-infective therapy. Her neurological status progressively improved, and the pregnancy was continued under maternal and fetal surveillance to 37 weeks of gestation. Because of residual motor impairment, reduced mobility, persistent sphincter dysfunction, and concern regarding effective expulsive efforts, cesarean delivery was selected. General anesthesia was chosen following multidisciplinary discussion. A healthy male infant weighing 3100 g was delivered, with Apgar scores of 10/10. At six weeks postpartum, the patient had achieved near-complete motor recovery, with autonomous walking, marked improvement in motor and sensory function, and almost complete resolution of sphincter dysfunction.

This case illustrates the importance of early diagnosis, multidisciplinary coordination, and appropriate neurological and obstetric management. Favorable maternal and neonatal outcomes may be achieved despite severe neurological impairment at presentation.

## Introduction

Transverse myelitis (TM) is a rare immune-mediated inflammatory disorder of the spinal cord [1]. It can result in sensory, motor, and autonomic deficits of varying severity [1]. Parainfectious TM (PITM) refers to spinal cord inflammation occurring in temporal association with an infection, most commonly of viral or bacterial origin [2].

Beyond a parainfectious origin, TM may occur following vaccination or in association with autoimmune diseases, such as systemic lupus erythematosus, as well as inflammatory or demyelinating disorders of the central nervous system, including multiple sclerosis, neuromyelitis optica spectrum disorder (NMOSD), and myelin oligodendrocyte glycoprotein antibody-associated disease (MOGAD). Less commonly, TM may be associated with certain drugs or toxic agents, or occur in a paraneoplastic context [3].

Pregnancy is characterized by dynamic adaptations of both innate and adaptive immunity that promote maternal tolerance toward the semi-allogeneic fetus, while maintaining immune competence. These changes evolve throughout gestation rather than representing a state of generalized immunosuppression [4]. The occurrence of acute TM during pregnancy is uncommon, and presents additional clinical challenges. Diagnostic evaluation must distinguish inflammatory myelopathy from compressive, infectious, autoimmune, and other demyelinating causes. Therapeutic decisions must simultaneously consider maternal neurological status and fetal well-being. Persistent neurological deficits and associated functional limitations may influence obstetric management [5]. In patients with spinal cord disorders, anesthetic planning, particularly the use of neuraxial techniques, requires individualized assessment of the underlying neurological condition [6].

We report the case of a 24-year-old woman who developed severe acute TM at 30 weeks of gestation in a febrile and inflammatory context suggestive of a parainfectious etiology. The case illustrates the diagnostic uncertainty that may persist, despite an extensive etiological workup, as well as the therapeutic, obstetric, and anesthetic decisions required when significant neurological impairment develops during the third trimester. It also documents continuation of the pregnancy to 37 weeks and progressive neurological improvement extending into the postpartum period.

## Case presentation

A 24-year-old gravida 3, para 2 woman, with no significant medical or surgical history, was admitted at 30 weeks of gestation for an acute neurological syndrome. The pregnancy had been uneventful until then. Symptoms developed rapidly, beginning with a sensation of heaviness, followed by progressive functional impairment of all four limbs, sensory disturbances, sphincter dysfunction, and mild respiratory discomfort, in the context of an unquantified fever. She was admitted on day 1 of symptom onset, and the neurological deficits progressed to their nadir within the first 24 hours. There were no visual disturbances, altered consciousness, or seizures.

On admission, the patient was conscious, afebrile, and hemodynamically stable. Her respiratory rate was approximately 19 breaths/min, with an oxygen saturation of 98% and no clinical respiratory instability. Neurological examination revealed asymmetric tetraparesis; the patient was unable to stand or walk. Muscle strength was graded 3/5 in the right upper limb, 1/5 in the right lower limb, and 4/5 in both the left upper and lower limbs. Muscle tone was decreased in all four limbs, deep tendon reflexes were brisk, and a right Babinski sign was present. Sensory examination demonstrated a T4 sensory level. Cranial nerve examination was normal, and higher neurological functions were preserved.

Spinal magnetic resonance imaging (MRI), performed on the first day of admission, demonstrated a T2-hyperintense lesion involving the anterior columns and extending longitudinally from C5 to T1 and transversely across more than half of the spinal cord circumference, with no evidence of an extrinsic compressive lesion (Figure 1). No associated intramedullary or perimedullary abnormalities were identified; the filum terminale and cauda equina nerve roots appeared normal. Brain MRI showed no abnormalities.

**Figure 1:**
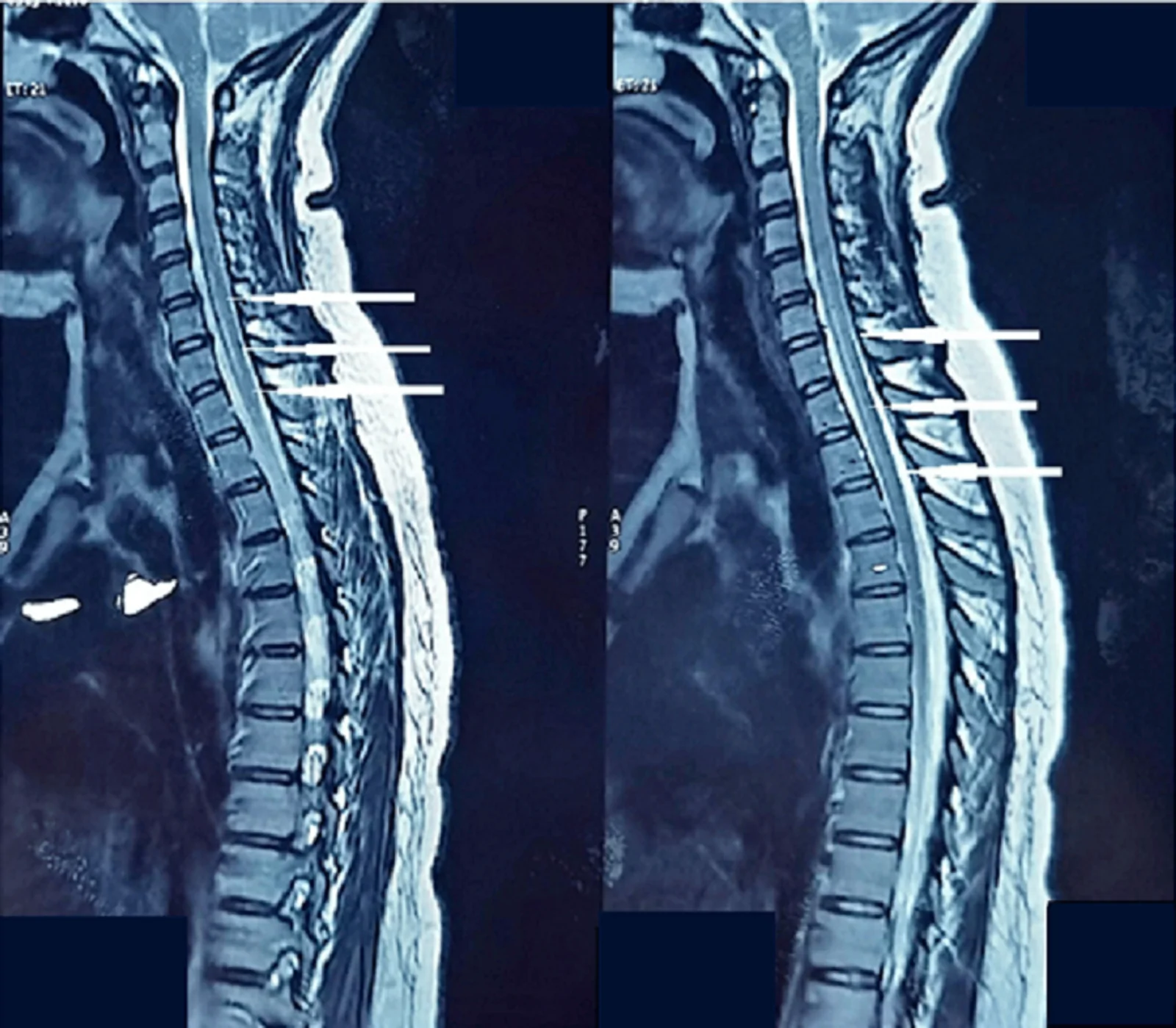
Spinal magnetic resonance imaging (MRI) showing a T2-hyperintense lesion involving the anterior columns, extending longitudinally from C5 to T1 and transversely across more than half of the spinal cord circumference (arrows)

Initial laboratory investigations showed mild systemic inflammation, with a C-reactive protein level of 11.60 mg/L and a white blood cell count of 12,900/mm³, including 80% neutrophils. Blood and urine cultures were negative. Serum testing for HIV, hepatitis A, B, C, and E, syphilis, cytomegalovirus, parvovirus B19, and herpes simplex virus was negative. Autoimmune investigations, including antinuclear antibodies (ANA), anti-aquaporin-4 (anti-AQP4) antibodies, and anti-myelin oligodendrocyte glycoprotein (anti-MOG) antibodies, were negative. No additional autoimmune antibody testing was documented in the available medical records. Initial renal function, electrolyte levels, and liver tests showed no significant abnormalities.

Cerebrospinal fluid (CSF) analysis showed 1 red blood cell/mm³, and 15 white blood cells/mm³ with lymphocytic predominance, consistent with lymphocytic pleocytosis. CSF protein was 0.184 g/L (laboratory reference range: 0.15-0.45 g/L), and CSF glucose was 0.59 g/L (laboratory reference range: 0.40-0.80 g/L). The IgG index was 0.65 (reference value <0.7), and isoelectric focusing of paired CSF and serum samples showed no evidence of intrathecal IgG synthesis. CSF PCR testing for HSV-1 and HSV-2 DNA was negative. Testing for anti-MOG and anti-neuromyelitis optica (anti-NMO) antibodies in the CSF was negative.

Based on the acute development of bilateral motor, sensory, and autonomic spinal cord dysfunction, the defined sensory level, progression to neurological nadir within 24 hours, exclusion of a compressive spinal cord lesion on MRI, the intrinsic spinal cord T2-hyperintense lesion, and CSF lymphocytic pleocytosis, the diagnosis of acute TM was established. A parainfectious etiology was considered because of the febrile onset, systemic inflammatory findings, and CSF lymphocytic pleocytosis. However, no specific infectious agent was identified.

Treatment was initiated within the first 24 hours of symptom onset. The patient completed a five-day course of intravenous methylprednisolone at 1 g/day. Acyclovir 250 mg every 12 hours and ceftriaxone 3 g every 24 hours were initiated empirically by the treating neurology team, in the setting of the initial febrile neurological presentation, while an infectious etiology was being investigated. Thromboprophylaxis with enoxaparin and functional rehabilitation were also initiated.

Liver enzymes were initially within normal limits, with an aspartate aminotransferase (AST) level of 17 IU/L and an alanine aminotransferase (ALT) level of 16 IU/L. Hepatic cytolysis was subsequently detected approximately 10 days after treatment initiation, and progressively worsened, reaching documented peak AST and ALT levels of 267 IU/L and 606 IU/L, respectively. Because methylprednisolone, acyclovir, and ceftriaxone had been administered concomitantly, the hepatic abnormalities could not be attributed to a specific medication. Treatment was temporarily discontinued, followed by a progressive decrease in liver enzyme levels. Following neurological reassessment, methylprednisolone was restarted at 240 mg/day and maintained until transfer to the obstetrics department at 37 weeks of gestation. By term, liver enzyme levels had markedly decreased, with AST and ALT levels of 34 IU/L and 38 IU/L, respectively. Corticosteroid therapy was progressively tapered during the postpartum period and ultimately discontinued.

Neurological improvement occurred progressively during the remainder of the pregnancy. Obstetric surveillance between 30 and 37 weeks included serial ultrasound assessment, with fetal biometric measurements appropriate for gestational age, fetal heart rate monitoring, and Doppler assessment, without evidence of fetal compromise or other specific obstetric complications.

At 37 weeks of gestation, residual motor impairment, reduced mobility, and persistent sphincter dysfunction remained. Because of these functional limitations and concern regarding the patient's ability to perform expulsive efforts during labor, the obstetric team elected to perform cesarean delivery.

The anesthetic approach was considered separately. Neuraxial anesthesia was discussed but was not selected. Following multidisciplinary discussion involving the obstetric, neurology, and anesthesiology teams, the anesthesiology team elected to perform the cesarean delivery under general anesthesia in the context of the recent TM and residual neurological deficits. No particular intraoperative hemodynamic or respiratory instability was reported, and no immediate postoperative neurological deterioration was observed.

A male infant weighing 3,100 g was delivered, with Apgar scores of 10/10. Immediate neonatal course was favorable. Maternal neurological improvement continued postpartum. At six weeks postpartum, the patient had achieved near-complete motor recovery, with autonomous walking, marked improvement in motor strength and sensory function, and almost complete resolution of sphincter dysfunction.

## Discussion

TM is an inflammatory disorder of the spinal cord resulting in motor, sensory, and autonomic dysfunction [1]. When it occurs following a viral or bacterial infection, it is referred to as PITM [2]. The annual incidence of TM is estimated at one to eight cases per million population [1]. Its age distribution is bimodal, with higher frequency during the second and fourth decades of life [3].

Its etiological spectrum includes infectious and parainfectious processes, systemic autoimmune diseases, multiple sclerosis, neuromyelitis optica spectrum disorder (NMOSD), myelin oligodendrocyte glycoprotein antibody-associated disease (MOGAD), drug- or toxin-related causes, and paraneoplastic disorders, while a proportion of cases remain idiopathic [3]. The occurrence of acute TM during pregnancy is uncommon, and published experience remains limited, particularly regarding diagnostic, therapeutic, obstetric, and anesthetic management [5,7-9].

The diagnosis in our patient was supported by the combination of an acute spinal cord syndrome with bilateral motor, sensory, and autonomic dysfunction, a defined T4 sensory level, progression to a neurological nadir within approximately 24 hours, and evidence of spinal cord inflammation. MRI demonstrated an intrinsic T2-hyperintense lesion extending from C5 to T1 while excluding an extrinsic compressive cause, and CSF analysis demonstrated lymphocytic pleocytosis. These findings are consistent with the diagnostic framework proposed by the Transverse Myelitis Consortium Working Group, which emphasizes clinically attributable spinal cord dysfunction, exclusion of compression, evidence of inflammation, and an acute-to-subacute temporal evolution [7]. Brain MRI was normal, anti-AQP4 and anti-MOG antibodies were negative, and the IgG index and isoelectric focusing provided no evidence of intrathecal IgG synthesis.

The etiology was less certain. The febrile onset, mild systemic inflammatory response, and CSF lymphocytic pleocytosis led to consideration of a parainfectious mechanism. However, no causative infectious agent was identified, including negative HSV-1 and HSV-2 PCR testing in the CSF. Consequently, a parainfectious origin should be regarded as suspected rather than established, and an idiopathic or other inflammatory/demyelinating etiology cannot be completely excluded. This distinction is important because TM encompasses a heterogeneous spectrum of infectious, immune-mediated, demyelinating, systemic autoimmune, and idiopathic etiologies [3,10].

Comparison with previously reported pregnancy-associated cases further illustrates the variability in clinical presentation and management. Hegde et al. described a 31-year-old primigravida who developed longitudinally extensive TM at 32 weeks of gestation following a febrile illness, with bilateral lower-limb weakness, sensory loss, bladder dysfunction, a C3-T2 lesion, and CSF lymphocytic pleocytosis. As in our patient, an extensive workup failed to identify a specific infectious agent, and a parainfectious mechanism was considered. Their patient received high-dose methylprednisolone, followed by intravenous immunoglobulin, and underwent emergency cesarean delivery at 35 weeks after developing urinary sepsis, with near-complete motor recovery at two months postpartum [8]. In contrast, our patient presented slightly earlier, at 30 weeks, with more extensive functional involvement including all four limbs, and pregnancy could be continued to 37 weeks without documented fetal compromise. She was managed with corticosteroid therapy, without additional IVIG or plasma exchange, and achieved near-complete motor recovery by six weeks postpartum.

Other published cases differ substantially in both gestational age and obstetric circumstances. Shekhar et al. reported acute TM presenting at 40 weeks with severe lower-limb weakness and inability to walk. Cesarean delivery in that case was performed emergently because of fetal distress and arrest of labor in the second stage, rather than because TM itself constituted an indication for cesarean delivery [9]. Conversely, women with preexisting TM have achieved term vaginal deliveries, supporting an individualized approach to the mode of delivery, rather than considering TM itself an indication for cesarean delivery [5]. In our patient, cesarean delivery was selected on an individualized functional basis: despite neurological improvement, residual motor impairment, reduced mobility, and persistent sphincter dysfunction raised concern regarding her ability to perform expulsive efforts during labor.

High-dose intravenous corticosteroids, most commonly methylprednisolone, are generally used as initial therapy for acute inflammatory TM, although treatment should ultimately be guided by the underlying etiology and clinical severity [10]. Our patient completed five days of intravenous methylprednisolone at 1 g/day. Acyclovir and ceftriaxone were administered empirically during the initial febrile presentation, while an infectious etiology was being investigated. The subsequent development of marked hepatic cytolysis complicated management, with documented AST and ALT peaks of 267 IU/L and 606 IU/L, respectively. Because several medications had been administered concomitantly, causality could not be assigned to a specific agent. Following temporary treatment interruption and improvement in liver enzyme levels, methylprednisolone was restarted at 240 mg/day and maintained until 37 weeks, followed by postpartum tapering. This prolonged and interrupted treatment course distinguishes our case from the pregnancy-associated cases discussed above [8,9] and illustrates the need to adapt neurological treatment to evolving maternal complications.

In patients with an inadequate response to corticosteroids, plasma exchange represents a therapeutic option [11]. During pregnancy, however, this treatment may expose the mother to complications, including hemodynamic instability, electrolyte disturbances, and coagulation abnormalities, as well as fetal complications, such as preterm uterine contractions or even acute fetal distress [12].

Because of the risks associated with plasma exchange during pregnancy, intravenous immunoglobulin (IVIG) has also been reported as an alternative, in selected cases of acute TM during pregnancy [12].

Anesthetic management represents a separate clinical issue from the indication for cesarean delivery. Obstetric patients with disorders involving the spinal cord require individualized anesthetic assessment, particularly when neuraxial techniques are considered, and the available evidence is largely derived from case reports, case series, and limited retrospective data [6]. Walsh et al. reported successful general anesthesia for cesarean delivery in a patient with idiopathic acute TM, and described anesthetic concerns including autonomic dysreflexia and hyperkalemia associated with succinylcholine [13]. Shekhar et al. also used general anesthesia for emergency cesarean delivery in a patient with acute TM, using a ProSeal laryngeal mask airway, without neuromuscular blockade [9]. Alternative anesthetic approaches have also been reported in obstetric patients with idiopathic TM [11]. Collectively, the limited available literature supports individualized anesthetic planning, rather than treating TM itself as an absolute indication for, or contraindication to, a particular anesthetic technique [6,9,11,13].

In our patient, the anesthesiology team selected general anesthesia, in the context of recent TM and residual neurological deficits. No particular intraoperative hemodynamic or respiratory instability or immediate postoperative neurological deterioration was reported.

The neurological evolution was ultimately favorable, despite substantial impairment at presentation. At 37 weeks, residual motor and sphincter dysfunction persisted, whereas by six weeks postpartum, the patient was walking independently, motor strength and sensory function had markedly improved, and sphincter dysfunction had almost completely resolved. However, this favorable evolution cannot be attributed solely to corticosteroid therapy on the basis of a single case. TM has a heterogeneous natural history, and neurological outcomes vary according to clinical, etiological, and radiological characteristics [14,15]. The clinical course should therefore be interpreted descriptively, rather than as evidence of a specific therapeutic effect.

Historically, untreated TM has been associated with an unfavorable prognosis, including mortality, persistent neurological sequelae, and progression to spastic paraplegia [16,17]. More recent data suggest that the prognosis of TM depends on the severity of the clinical and radiological spinal cord involvement: more severe neurological impairment at presentation and the presence of spinal cord abnormalities on MRI have been associated with less favorable outcomes [14]. Other data suggest that etiology may also have prognostic significance, with cases double-seronegative for anti-AQP4 and anti-MOG antibodies being associated with fewer relapses and better functional outcomes [15].

## Conclusions

Acute transverse myelitis during pregnancy is an uncommon condition. It poses diagnostic, therapeutic, obstetric, and anesthetic challenges. This case illustrates the management of severe third-trimester TM, with an uncertain etiology, requiring coordinated multidisciplinary care and individualized decisions regarding neurological treatment, continuation of pregnancy, mode of delivery, and anesthesia. Despite substantial neurological impairment at presentation, the pregnancy progressed to 37 weeks with a favorable neonatal outcome, while maternal neurological function progressively improved, with near-complete motor recovery, and almost complete resolution of sphincter dysfunction by six weeks postpartum.

## References

[1] West TW (2013). Transverse myelitis--a review of the presentation, diagnosis, and initial management. Discov Med.

[2] Kerr DA, Ayetey H (2002). Immunopathogenesis of acute transverse myelitis. Curr Opin Neurol.

[3] Beh SC, Greenberg BM, Frohman T, Frohman EM (2013). Transverse myelitis. Neurol Clin.

[4] Weng J, Couture C, Girard S (2023). Innate and adaptive immune systems in physiological and pathological pregnancy. Biology (Basel).

[5] Berghella V, Spector T, Trauffer P, Johnson A (1996). Pregnancy in patients with preexisting transverse myelitis. Obstet Gynecol.

[6] Walsh E, Zhang Y, Madden H, Lehrich J, Leffert L (2021). Pragmatic approach to neuraxial anesthesia in obstetric patients with disorders of the vertebral column, spinal cord and neuromuscular system. Reg Anesth Pain Med.

[7] Transverse Myelitis Consortium Working Group (2002). Proposed diagnostic criteria and nosology of acute transverse myelitis. Neurology.

[8] Hegde A, Rai AV, Shetty A (2026). Parainfectious transverse myelitis in a pregnant woman: a rare clinical entity. Cureus.

[9] Shekhar S, Kadian S, Sony S, Chaudhary S, Jakhar J (2022). Acute transverse myelitis in pregnancy: the use of ProSeal laryngeal mask airway without curarization for emergency cesarean section. Cureus.

[10] Tisavipat N, Flanagan EP (2023). Current perspectives on the diagnosis and management of acute transverse myelitis. Expert Rev Neurother.

[11] Saxena KN, Kaul A, Shakir M (2019). Anesthetic management of an obstetric patient with idiopathic transverse myelitis. A unique approach!. J Obstet Anaesth Crit Care.

[12] Connelly-Smith L, Alquist CR, Aqui NA (2023). Guidelines on the use of therapeutic apheresis in clinical practice - evidence-based approach from the Writing Committee of the American Society for Apheresis: The ninth special issue. J Clin Apher.

[13] Walsh P, Grange C, Beale N (2010). Anaesthetic management of an obstetric patient with idiopathic acute transverse myelitis. Int J Obstet Anesth.

[14] Zhou Y, Chen Q, Gan W (2024). Comparison between MRI-negative and positive results and the predictors for a poor prognosis in patients with idiopathic acute transverse myelitis. BMC Neurol.

[15] Rocchi C, Forcadela M, Kelly P (2024). The absence of antibodies in longitudinally extensive transverse myelitis may predict a more favourable prognosis. Mult Scler.

[16] Abboud H, Sun R, Modak N, Elkasaby M, Wang A, Levy M (2024). Spinal movement disorders in NMOSD, MOGAD, and idiopathic transverse myelitis: a prospective observational study. J Neurol.

[17] Andrianakos AA, Duffy J, Suzuki M, Sharp JT (1975). Transverse myelopathy in systemic lupus erythematosus: report of three cases and review of the literature. Ann Intern Med.

